# DynTex: A real-time generative model of dynamic naturalistic luminance textures

**DOI:** 10.1167/jov.25.11.2

**Published:** 2025-09-03

**Authors:** Andrew Isaac Meso, Jonathan Vacher, Nikos Gekas, Pascal Mamassian, Laurent U. Perrinet, Guillaume S. Masson

**Affiliations:** Neuroimaging Department, Institute of Psychiatry, Psychology and Neuroscience, King’s College London, UK; MAP5, Université Paris Cité, CNRS, Paris, France; Department of Psychology, School of Applied Sciences, Edinburgh Napier University, Edinburgh, UK; Laboratoire des Systèmes Perceptifs, Département d'études cognitives, École normale supérieure, PSL University, CNRS, Paris, France; Institute de Neurosciences de la Timone, Aix-Marseille Univ, CNRS, Marseille, France; Institute de Neurosciences de la Timone, Aix-Marseille Univ, CNRS, Marseille, France

**Keywords:** Motion Clouds, dynamic textures, visual perception, GPU, psychophysics

## Abstract

The visual systems of animals work in diverse and constantly changing environments where organism survival requires effective senses. To study the hierarchical brain networks that perform visual information processing, vision scientists require suitable tools, and Motion Clouds (MCs)—a dense mixture of drifting Gabor textons—serve as a versatile solution. Here, we present an open toolbox intended for the bespoke use of MC functions and objects within modeling or experimental psychophysics contexts, including easy integration within Psychtoolbox or PsychoPy environments. The toolbox includes output visualization via a Graphic User Interface. Visualizations of parameter changes in real time give users an intuitive feel for adjustments to texture features like orientation, spatiotemporal frequencies, bandwidth, and speed. Vector calculus tools serve the frame-by-frame autoregressive generation of fully controlled stimuli, and use of the GPU allows this to be done in real time for typical stimulus array sizes. We give illustrative examples of experimental use to highlight the potential with both simple and composite stimuli. The toolbox is developed for, and by, researchers interested in psychophysics, visual neurophysiology, and mathematical and computational models. We argue the case that in all these fields, MCs can bridge the gap between well- parameterized synthetic stimuli like dots or gratings and more complex and less controlled natural videos.

## Introduction

Any scientific attempt at understanding vision, or any other sensory modality, from an ecological perspective would typically start with a precise characterization of the relevant physical properties of the environment. From there, the next step is to measure the quantitative relationships between these physical properties and the behavior of the organism in order to elucidate the computational rules governing visual information processing (Marr, 2010). Visual psychophysics investigates this relationship in the context of human perception. Since the pioneering work of Fechner, Weber and Wundt on psychophysical methods and their theoretical foundation, a variety of psychophysical tasks have been elaborated in humans (see Kingdom & Prins, 2016; Pelli & Farell, 1999) and eventually shared with biologists who investigate visual behaviors and their underlying neural bases. The ability of most animals to measure and process dynamic information about their ever-changing environment has made visual motion studies a landmark of this approach.

### A legacy of simple motion stimuli

A critical step along this path of designing functional tasks is the definition and generation of well-controlled, calibrated visual stimuli. In visual psychophysics and neuroscience, stimuli are most often rooted in the theoretical characterizations of particular physical properties of the visual environment (a generative model) and the corresponding theoretical assumptions about how biological visual systems would extract and represent visual information (a decoding model). For instance, luminance-defined sinusoidal gratings, and their various derivatives, are grounded on the idea that visual stimuli are best described by their Fourier properties and that early stages of the visual system act as frequency analyzers (Campbell & Robson, 1968; Graham, 1989). Since then, multiple variants of grating combinations have been proposed to probe motion processing for simple-to-complex motion patterns, the best-known example being moving plaids made by adding two different drifting gratings (Adelson & Movshon, 1982).

Random dot patterns constitute another class of stimuli used for investigating motion perception (Marshak & Sekuler, 1979). A variety of such stimuli have been designed over the last four decades, from single-direction random dot kinematograms (RDKs) to complex velocity flow fields. The main objective was to provide motion information without introducing specific spatial moving features (e.g., Braddick, 1974). A second objective was to control the position of every local motion element in both space and time while manipulating the signal-to-noise ratio (SNR) (e.g., Nawrot & Sekuler, 1990). Computer-generated velocity flow fields could be designed to render the optic flow patterns produced on the retina by the relative displacement between our eyes and the three-dimensional (3D) environment (van Doorn & Koenderink, 1982). Their popular usage in both visual motion psychophysics and neurophysiology reinforces the modular hypothesis that a dedicated set of local detectors could sense motion stimuli to extract speed and direction information independently of any other image properties (see Nishida, 2011, for a review).

Gratings and RDKs have been the two Janus faces of motion stimuli. The two classic types have a highly contrasted spatial structure most evident in their respective spatial frequency content. The first has a point-like energy spectrum in Fourier space while the second has a broad-band spectrum. Gratings have a homogeneous modulation in space-time while RDKs can be finely manipulated over both space and time. These two classes of stimuli have had a profound influence on our theoretical perspectives about how visual motion is computed by biological systems. For instance, they shaped the standard feedforward, two-stage computational framework of biological motion processing, where a first stage of spatiotemporal filters is followed by a second, pooling stage that encodes speed and direction of global motion (Simoncelli & Heeger, 1998). Interestingly, the receptive field properties of a putative neural implementation of these two stages, the primary visual cortex (V1) and middle temporal (MT) areas of primates, were predominantly investigated using either one or the other stimulus class. They were pivotal in identifying the key computational steps of motion detection and integration stages (Rust & Movshon, 2005). However, at the perceptual level, these simple stimuli have inadvertently curbed our understanding of motion perception, resulting in a restricted insight into the problem of computing the global velocity field of natural images (see Nishida, Kawabe, Sawayama, & Fukiage, 2018, for a review). Indeed, very few neurophysiological studies have directly compared neuronal responses of the same cells (or areas) to gratings and RDKs or a combination of both (Xiao & Huang, 2015), and the attempts at reconciling and modeling how these cells can change properties when presented with either of the two classes remain rather limited (Medathati, Rankin, Meso, Kornprobst, & Masson, 2017).

### Bridging the natural and the artificial: Naturalistic Motion Clouds

More recently, two novel classes of moving stimuli have been proposed to overcome these intertwined limitations. First, in the last two decades, natural images and movies have become increasingly popular to investigate visual processing (Simoncelli & Olshausen, 2001). However, when designing perceptual tasks, their richness in terms of diversity of objects and motion signals is inherently limited by their lack of experimental control and perceptual complexity (Hénaff, Goris, & Simoncelli, 2019). This search for more realistic stimuli led to seminal attempts to generate artificial stimuli that render specific appearances of natural scenes, such as animacy or transparency (Kawabe, Maruya, Fleming, & Nishida, 2015). Second, filtered RDKs or micro-patterns where single, pixel-like elements of a sparse array of local motions are replaced with Gabor patches have been used to probe the spatiotemporal properties of motion integration and segmentation (Amano, Edwards, Badcock, & Nishida, 2009; Rider, McOwan, & Johnston, 2009). These novel stimuli stress the growing need for naturalistic stimulation where high complexity and dimensionality remain fully tractable and controllable, thanks to suitable generative models of natural scenes statistics in both space-time and Fourier domains.

**Figure 1.**
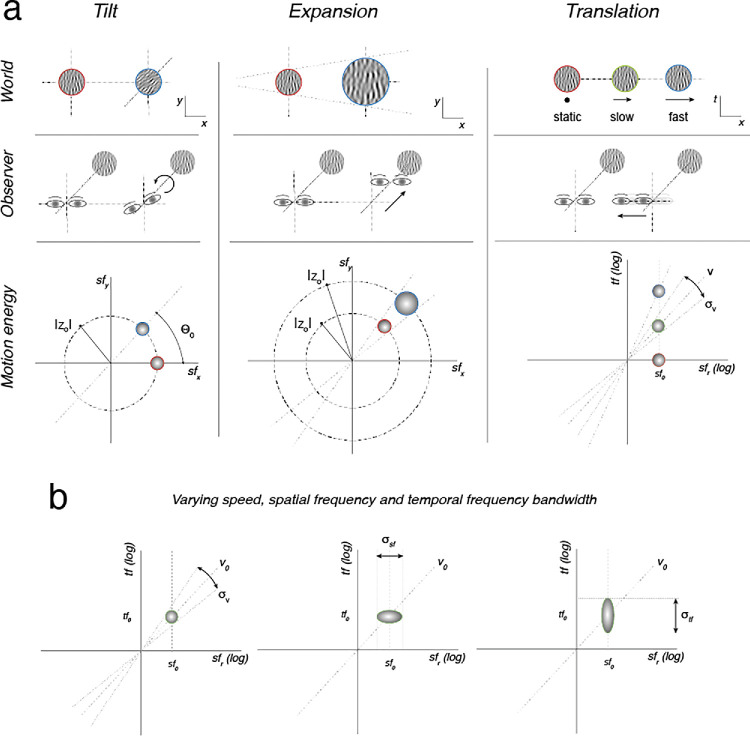
Different moving patterns of Motion Clouds can be derived from a generative model of an observer’s movement in a textured environment. (a) A change in orientation (left), size (center), or velocity (right) corresponds to a rotation or a forward or a lateral translation of the observer, respectively. These changes in the viewing point over time can be modeled within the Fourier space to produce different Motion Clouds. (b) Within this framework, mean and variance of each stimulus parameter (orientation, spatial frequency, speed, temporal frequency) can be manipulated (Gekas, Meso, Masson, & Mamassian, 2017; Vacher et al., 2015). In the plots: *x* stands for horizontal space, *y* vertical space, *t* time, *sf* spatial frequency, *tf* temporal frequency, *v* speed, and σ bandwidth.

To meet these objectives, we previously proposed a new class of moving stimuli called “Motion Clouds” (MCs) (Leon, Vanzetta, Masson, & Perrinet, 2012; Vacher, Meso, Perrinet, & Peyré, 2015; Vacher, Meso, Perrinet, & Peyré, 2018). They are characterized by their power spectrum, that is, by how energy is distributed across spatial and temporal frequencies. In practice, they are generated as dynamic random phase textures (Galerne, Gousseau, & Morel, 2010; Leon et al., 2012) that are optimal artificial stimuli for the study of luminance-based visual processing. Our initial implementation provided experimenters with a fine control of first- and second-order stimulus statistics (i.e., mean and variance along each stimulus dimension), as Gaussian-like distributions in the spatiotemporal frequency space. Alternatively, but equivalently, Motion Clouds can be formulated as a linear combination of an infinite set of spatiotemporal Gabor kernels parameterized by their scale, orientation, direction, and speed distributions directly in the spatiotemporal domain (Galerne, Gousseau, & Morel, 2010; Vacher, Meso, Perrinet, & Peyré, 2018); see Figure 1. A theoretical consideration of the equivalence of these approaches was necessary to properly characterize the counterpart of the speed distribution used in the original work for use with the kernels in the spatiotemporal frequency domain. These MCs are naturalistic in the sense that they can be manipulated in both space and time to simulate different changes of the observer’s view points, as illustrated in Figure 1a. Different moving patterns can be precisely generated to correspond to, for instance, a tilt, a forward displacement, or a translation of the observers relative to the environment in which visual statistics are defined. Although MC stimuli were originally defined as global motion patterns that could model ecological rotation, looming, and translation movements like those illustrated in the figure, more complex two-dimensional (2D) and 3D patterns can be generated from them, thanks to their texture elements that can be globally and individually (e.g., spatial distribution) controlled.

### Advances in precise control of second-order statistics and fast computation

From the legacy of (moving) visual stimuli, we learned that three factors are important when designing and disseminating visual stimulation tools. First, stimulus parameters should encapsulate the versatile set of features that a given visual system is sensitive to. Second, relevant parameters should be effectively modifiable and controllable by experimenters. Third, all of this should be achieved in a user-friendly and accessible way, in the spirit of open-science. Our previous work achieved the first of these aims: producing a stimulus that was more naturalistic and versatile than gratings, dots, or their composites (Leon et al., 2012; Vacher et al., 2018). The stimuli were closer to natural scenes when compared to dots and gratings in their broadband frequency distributions and 1/*sf* frequency power spectrum (Field, 1987). The current technical contribution seeks to strengthen the last two aspects by providing more clearly documented access to Motion Cloud stimuli and producing an intuitive understanding of stimulus parametric control in a user-friendly, open-source, and easily accessible toolbox called DynTex.

When studying visual motion, it is often critical to vary the strength of the stimulus. In RDKs, this is usually done by varying the coherence of the dots moving in one particular direction. Such stimulus strength decreases when either the fraction of dots moving in random directions (or speeds) is increased or when the spread of the distribution of dot motion directions (or speeds) is increased. In moving gratings, motion strength can be manipulated by varying the stimulus contrast, although contrast usually has a limited effect on performance for suprathreshold stimuli. In natural movies, stimulus strength is sometimes manipulated by superimposing multiple movies, although this creates some ambiguity about which movie the observer should attend to.

The MC stimulus has a natural set of parameters that control its strength. These parameters are the bandwidths in spatial frequency, orientation, direction, and speed (see Figure 1b) . These bandwidth parameters allow users to continuously control the characteristic frequency envelope of the stimulus, going from a single point in frequency space that makes the stimulus similar to a grating, to a broadband spectrum that makes the stimulus more similar to (filtered) random dots. For instance, the speed bandwidth controls the extent to which the stimulus contains a single speed such that a narrow bandwidth causes a stimulus similar to the translation of a rigid object, while a large bandwidth would mix a range of speeds that would be characteristic of nonrigid moving objects or several transparent objects. RMS contrast, which drives perceived contrast, can also be directly manipulated by setting the variance of the pixel value distribution. These second-order stimulus statistics are good candidates for control of input strength.

**Figure 2.**
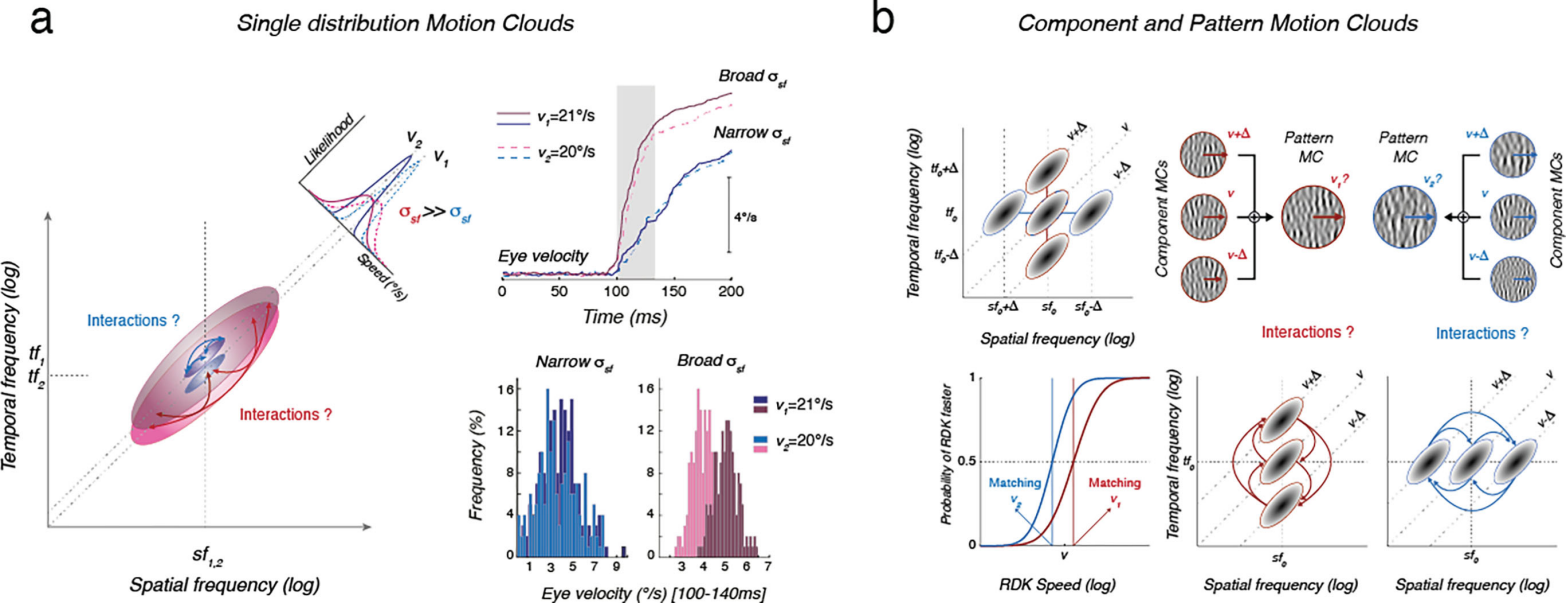
Two illustrative examples of previous uses of Motion Clouds in published work and resulting key findings. (a) Motion Clouds moving at two different, nearby speeds (20 and 21°/s) are presented at either small or large spatial frequency bandwidths. They trigger reflexive tracking eye movements, called ocular following responses. Distributions of initial eye velocity across trials are plotted for each condition. At small bandwidths, there is no difference between responses to either 20 or 21°/s (light and dark blue). At large bandwidths, responses between the two speeds are separated, indicating that speed is better estimated and responses are more reliable across trials. Thus, speed is more precisely estimated when energy is largely distributed along the velocity plane (Simoncini et al., 2012). (b) Subsequent psychophysical experiments compared speed discrimination performance for Motion Clouds called patterns or triplets, which are made of three component MCs whose relative positions are manipulated around a central, reference component. Speed discrimination performance changes depending upon the orientation of the triplets. From these perceptual biases, one can infer the pattern of excitatory and inhibitory interactions between channels paving the spatiotemporal Fourier plane (Gekas et al., 2017).

Over the last decade, we demonstrated the power of this approach to probe visual motion processing in the context of either motion perception or tracking eye movements in humans (Gekas et al., 2017; Meso, Gekas, Mamassian, & Masson, 2022; Simoncini, Perrinet, Montagnini, Mamassian, & Masson, 2012; Vacher et al., 2018). In short, these different bandwidth parameters control the amount of task relevant information within the sensory input and thus impacts our ability to discriminate and estimate several important motion features such as direction or speed, as illustrated in Figures 2a, 2b. They can also change stimulus appearance. For instance, simple or complex MCs can be generated. In the first case, the statistics of a single envelope is manipulated (Figure 2a). In the second case, several Motion Clouds can be generated with different properties and combined, similar to the classic component and pattern approaches with gratings and plaids (Figure 2b). This allows users to probe the interactions between the channels sensing each component within the spatiotemporal frequency space. One second working hypothesis is that these different bandwidths explicitly encode the accuracy of a visual stimulus. We tested this hypothesis by recording neural activity in the primary visual cortex of cats (Ladret et al., 2023). We showed that the precision of neural responses can be affected by changing the orientation bandwidth. Similar results were obtained with direction and speed selectivity of retinal ganglion cells in rodents (Ravello, Perrinet, Escobar, & Palacios, 2019). Overall, psychophysical and physiological studies highlight the fact that manipulating the different bandwidths of MC offers interesting prospects for studying the spatial and temporal integration of visual information for different dimensions, such as orientation or motion processing.

Our earliest implementation of MC tools used stimulus generations directly carried out in Fourier space where mean frequency and bandwidth envelope parameters could be specified. The output was fed into a fast Fourier transformation (FFT) to project these envelopes into image space (Leon et al., 2012). This sequence of computational steps had the advantage of being mathematically exact, but stimulus spatiotemporal matrix dimensions had to be predefined and the subsequent processes involved computationally intensive FFT steps. For a 256-by-256 image sequence, for example, to have comparable frequency resolution across space and time for generation, the temporal dimension would also be 256 frames. Such a large matrix has nonlinear effects on memory use and takes several seconds to generate for a high spec computer. When the image size is scaled to 512 × 512 and the number of frames to 512, processing load rises at best as a cubic function, extending generation into minutes. Subsequently, we developed a new implementation based on an autoregressive algorithm (AR2), which generates stimuli of specified parameters on a frame-by-frame basis and uses numerical calculus methods to finely control spatiotemporal correlations across the frames (Vacher et al., 2015; Vacher et al., 2018). There is an algorithmic difference in these processes that means the dimensionality of generation matrices is cubic and an order of magnitude larger for the FFT than the square matrices in AR2, and this is further explained in subsequent technical sections. This technique, which we exploit in the new DynTex toolbox, has the advantage of enabling the real-time generation of motion stimuli and thus opens up the possibility of continuously varying parameters during MC generation. Implementation of the intensive matrix operations on the GPU, coupled with use of GPU graphics display processing during experiments conducted with either Psychtoolbox or PsychoPy environments (Kleiner, Brainard, & Pelli, 2007; Peirce et al., 2019), will help maximize the potential for its empirical use across a broad range of applications in vision science.

There are several existing toolboxes already available for visual motion psychophysics that allow users to generate, view, and store classic stimuli such as random dot kinematograms, gratings, Gabors, and natural images, including implementations in Psychtoolbox and PsychoPy (Kleiner, Brainard, & Pelli, 2007; Peirce et al., 2019). There are, however, limited options when it comes to dynamic textures.

Spatiotemporal luminance noise does not lend itself well to full parametric control, so standardized approaches are needed for reproducible and transparent research. Here, we describe a single package (DynTex) that can be used to generate, display, and store Motion Clouds with well-controlled, screen-based spatiotemporal parameters. The online generation of usable noise matrices involving frequency manipulation requires fast computations, and in the past, this has involved the use of executables from precompiled programming languages like C/C++, which are more efficient with memory allocation and use (e.g., Meso & Hess, 2010; Meso & Hess, 2012; Watson & Eckert, 1994). As far as we know, for vision science, the proposed approach is the fastest technique for generating real-time MCs with large arrays (e.g., 512 by 512) at high refresh rates (as tested up to 120 Hz so far). This has been implemented in higher-level languages (MATLAB and Python) that are widely used within the visual neuroscience community. To maximize reach and use of the toolbox, this article provides examples of illustrative task code using Psychtoolbox to demonstrate bandwidth parameter manipulation, multicomponent MCs and millisecond generation during intertrial intervals of the task flows. The inclusion of a graphic user interface (GUI) will enable users to easily visualize how changes in the parameter space alter both the appearance of the moving images and their spectral characteristics. We recommend adding this DynTeX toolbox to experimenters’ repositories alongside other tools (e.g., Kleiner, Brainard, & Pelli, 2007; Peirce et al., 2019) as an accessible window into the powerful world of dynamic texture-based generative models of vision.

## Methods

### Defining Motion Clouds

#### Speed distribution and power spectrum

The power spectrum of static natural images has long been characterized (Field, 1987). It is known to decrease radially as 1/*sf*, the inverse of spatial frequency, and to have more energy around the vertical and horizontal orientations (Wainwright, Schwartz, & Simoncelli, 2002). The relationship between the scale and orientation distributions and the power spectrum is precisely established and can be read directly from the power spectrum of static images. In comparison, the power spectrum of natural movies is hypothesized to reflect the distribution of speed of the moving objects they contain (Dong & Atick, 1995). While it is accepted that the speed is a function of the ratio between temporal and spatial frequencies, it remains unclear if such a function corresponds to naturalistic speed distributions. MCs were originally conceived to study dynamic visual processing under more natural broadband power spectrum stimuli (Leon et al., 2012). Therefore, a key element was the parametrization of speed distributions in the spatiotemporal power spectrum. To solve this question, MCs were redefined as a random aggregation of localized drifting Gabor elements *G*_*i*_ of characteristics *C*_*i*_ (defining orientation θ_*i*_, spatial frequency *sf*_*i*_), which can be formulated as

(1) $$\begin{array}{c} \;I\left(x,y,t\right)=\frac{1}{\lambda }\sum_{i=1}^{+\infty }a_{i}G_{i}\left(x-x_{i}-{v_{x}}_{i}t,y-y_{i}-{v_{y}}_{i}t,C_{i}\right),\; \end{array}$$

where λ is the intensity of the Poisson process controlling the number of random positions (*x*_*i*_, *y*_*i*_). Using this definition allows one to properly define the distribution of speed *v* = (*v*_*x*__*i*_, *v*_*y*__*i*_) (and characteristics *C*_*i*_). As the intensity λ tends toward infinity, the image *I* becomes a stationary Gaussian process with a power spectrum that can generate a transformation of the speed distribution. In the case where *v* = *v*_0_ + δ*v* with *f*_δ*v*_ being the distribution of ||*v*||, the transform produces for a real number *u*,

(2) $$L\left(f_{\delta v}\right)\left(u\right)=\int_{-\pi }^{\pi }f_{\delta v}\left(-\frac{u}{\cos(\theta )}\right)d\theta .$$

We refer to Vacher et al. (2018) for the complete derivation of this result. Key to the derivation is the relationship between the transform of a Gaussian distribution and the reverse transform of the distribution. The reverse transform of the temporal profile of the AR process is crucial for the fully controlled generation of MCs.

#### Parameterized spectral characteristics

The MCs are fully characterized by parameters *M* and *U*, which define the aggregate characteristics *C*_*i*_ of orientation, spatial frequency, and speed initially set for Gabor elements in Equation 1,

(3) $$M=\left[\theta_{0},sf_{0},v_{0}\right]\;\text{and}\;U=\left[\Delta \theta ,Bsf,Bv\right],$$

where *M* sets the central tendency characteristics of these parameters and *U* their spread. The envelope probability distribution *E* is therefore constructed from the statistics as

(4) E=P[M,U].

Equation 4 is the power spectrum function with the stimulus central tendency *M* and spread *U* specified. The orientation follows a von Mises distribution while the spatial frequency follows a log-Normal distribution, and the speed distribution is obtained from the reverse transform of the function of the temporal and spatial frequency ratio that appears in the power spectrum (see Vacher et al., 2018, for details). In Motion Clouds, this reverse transform is specified by the autoregression used to generate the frames in real time.

### Autoregression

In order to generate stimuli continuously using an autoregressive algorithm, one can use a stochastic partial differential equation (sPDE) formulation to derive causal equations, that is, equations that constrain dynamic correlations into the future, but at the same time remain local in time (Vacher et al., 2018). These were derived as so-called generalized stochastic processes so that linear transformations like differentiation and Fourier transformations can be applied (Unser, Tafti, & Sun, 2014). Looking briefly at this derivation, we consider translation along the horizontal (*x*) direction only and create the sPDE cloud *I* from another cloud *I*_0_, which does not have a translational speed. In this case, *I*_0_ is a stationary Gaussian field such that the global frequency statistics remain the same across the stimulus defined by Equation 3:

(5) $$I\left(x,y,t\right)=I_{0}\left(x-v_{0}t,y,t\right).$$

To maintain such stationarity, spatial and temporal covariance ∑_*W*_ is controlled by filters (α, β) and a translation speed *v*_0_. The driving noise generating the random field *D*(*I*_0_) is white in time and has a two-dimensional stationary covariance σ_*W*_.

(6) $$D\left(I_{0}\right)=\frac{\partial^{2}I_{0}}{\partial t^{2}}+\alpha *\frac{\partial I_{0}}{\partial t}+\beta *I_{0}.$$

**Figure 3.**
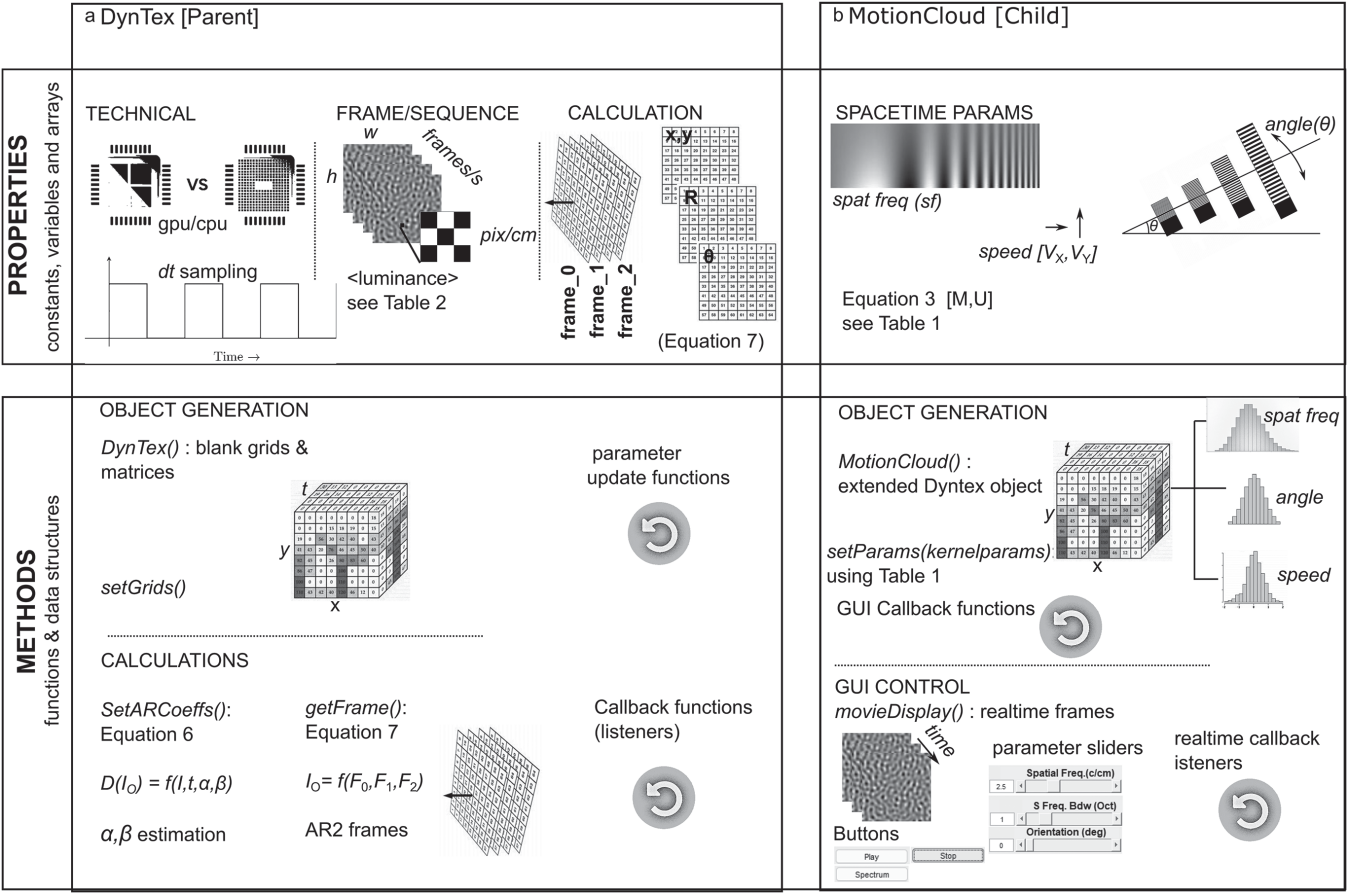
The properties and methods of the parent and child objects used to generate AR Motion Clouds. (a) General parent Dyntex object showing illustrations of the generation constants, variables, and arrays on the top and the matrices and calculations on the bottom. (b) The specific case of the child Motion Cloud object showing illustrations of the parameters detailed in Table 1 on the top and generation processes including the graphic user interface generation (GUI) on the bottom.

The filters (α, β) enforce additional temporal correlations of the model over time. Equation 6 can be recast in the Fourier domain, where a set of independent stochastic ordinary differential equations of the frequencies is equated to the spatial power spectrum of the driving noise and solved to find stationary solutions forward in time *t*. The solutions provide numerical equations that can be used to generate discrete sets of 2D frames separated by a time-step Δ, which estimate the first and second derivative terms. The complete derivation and associated proofs are beyond the scope of this article but are detailed in our more theoretical publication (Vacher et al., 2018). The crucial result for this numerical simulation of MCs is the recursive expression that follows:

(7) $$\begin{array}{ccc} \;I_{0}^{\left(l+1\right)} & = & \left(2\delta -\Delta \alpha -\Delta^{2}\beta \right)*I_{0}^{\left(l\right)}\\ & & +\;\left(-\delta +\Delta \alpha \right)*I_{0}^{\left(l-1\right)}+\Delta^{2}W^{\left(l\right)},\; \end{array}$$

where 2δ is a 2D Dirac delta distribution, and (*W*^(*l*)^)_*l*_ are 2-dimensional Gaussian fields and (*l* − 1), (*l*) and (*l* + 1) are recursively generated frames that contribute to making Equation 7 a steady-state solution to the original sPDE, so that after an initial warm-up phase of frame generation, the process will reach time stationarity, a convergence that typically takes under 100 frames. The convolutions on individual frames are carried out with fast Fourier transforms using the GPU where available. Within the code, the iterative generation across three frames implemented by Equation 7 can be seen in Figure 3a, under the calculations in the properties and methods.

### Key DynTex inputs and variables

The DynTex toolbox generates MCs with user-specified spatiotemporal parameters. There are several quantitative constants and variables that are needed for generation. In Table 1, we list the six key spatiotemporal parameters covering the envelope central tendency and spread characteristics of orientation, spatial frequency, and speed for the MCs and the corresponding in-code variable names. Note that the code variable labels th, sf, and speed from the table correspond to θ_0_, *sf*_0_, and *v*_0_ from Equation 7. Similarly, their distribution parameters th_sig, sf_bdw, and speed_sig correspond to characteristics specified by M in the same equations, Δθ, *Bsf*, and *Bv*. These parameters are all controlled by the object generation code of the Motion Cloud object in Figure 3b. The table also includes internally generated variables transformed from the six to represent temporal characteristics (*tf*) or speeds in output screen coordinates.

**Table 1.** Key spatiotemporal parameters for MC generation with default values and notes on generation.

Equation	In-code variable	Default	Notes
(3)	th	0	Vertical orientation
(3)	sf	2.5	Cycles per degree
(3)	speed	[0,0]	Direction and zero mean speed
(3/4)	th_sig	10	Sigma/bandwidth degrees
(3/4)	sf_bdw	1.0	Default octave bandwidth
(3/4)	speed_sig	50	Sigma/bandwidth pix/sec
N/A	speed_pxpf	Variable	Pix per frame screen adjusted
N/A	speed_sig_pxpf	Variable	Screen adjusted
(5/6)	tf	Internally calculated	Cycles per S

When using the generated MCs in real experiments or theoretical simulations, the user can precisely control the output screen calibration parameters. The variables controlling these settings can be found in the object-generating code of Figure 3b with details given in Table 2. MCs are generated in a square with lengths $siz\_im=2^{n}$, where *n* is typically a power of 2 (e.g., 7, 8, 9, or 10), so that the corresponding square image dimension siz_im has values of 128, 256, 512, or 1,024. During generation, this image dimension siz_im corresponds to the size of the image matrix terms *I* and *I*_0_ in Equations 1, 5, and 7. The setting of frames per second could be adjusted for typical psychophysics screens like the Cambridge Research Systems 32-inch Display++, which has a default refresh rate of 120 Hz and screen pixel sizes corresponding to 27.1 pixels per centimeter at a viewing distance of 57 cm. We tested the Dyntex toolbox on several computers with MathWorks MATLAB versions 2019–2023, using multi-core processors like the Intel Xeon or Core i7/i9 with maximum clocking speeds of 3.0G Hz or more. We typically had at least 16 MB of RAM and 4 GB NVIDIA graphics cards. We recommend these specifications as minimal requirements for a good performance of the DynTex toolbox. More systematically, we test MC production for a range of parameters on a high spec PC in a university computer lab and compare results with a Macbook M2 Pro in the next section.

**Table 2.** Additional parameters for setting stimulus physical sizes and refresh rates and then calibrating generation, display, and experimental output units.

In-code variable	Default	Purpose
siz_im	256	Image *x* and *y* lengths in pixels
fps	50	Frames per second (Hz)
px_per_cm	50	Pixels per screen cm
contrast	35.0	RMS contrast (pixel SD)
octa	1	Bandwith in octaves (1) or cyc/im (0)

The key output of the MC generation is a three-dimensional array with two spatial dimensions *x* and *y*, and a temporal dimension *t*. The square image has a fixed size in pixels of x=y=siz_im, while the number of frames generated will determine the length of the number of frames along *t*. These output frames form part of the image sequence matrix *I*_0_ in Equation 7 and are initiated within the object generation methods of the Dyntex and Motion Cloud objects; see Figures 3a, b. One of the advantages of the DynTex toolbox is that any number of frames can be generated continuously in real time.

## DynTex toolbox components

### Two object classes Dyntex and Motion Clouds

The source code of the toolbox was implemented with an object-oriented approach producing a modular structure that allows the user to generate and manipulate multiple instances of Motion Clouds in real time. There are two classes, the first of which is DynTex, the parent. This is a general dynamic texture class that starts in its properties by predefining some technical and calculation attributes for the generation of instances of Motion Clouds. The generation is carried out by the GPU by default, but if the computer and program in question cannot access the GPU, the CPU will be used. Stimulus size, refresh rate, and display calibration parameters as given in Table 1 are preset during initialization. The methods within this class are separated into object generation and calculation functions. The object generation presets working grids to preallocate memory either for GPU or CPU computations. The calculation of key AR coefficients from Equations 6 and 7 is carried out by a set of functions illustrated on the left half of Figure 3. DynTex contains a number of callback and listener functions, which react to the updating of generating parameters and adjust variables to let users change parameters in real time.

The child class, Motion Cloud, generates an autoregressive image sequence of a specific MC. The class properties are used to initiate the onset of spatiotemporal parameters, as specified by Equations 3 and 4 and listed in Table 1. The methods in this class first call on the Dyntex parent class using the grids and technical presets (object generation see Figure 3a) to apply the spatiotemporal distribution parameters that characterize the given MC object (child object generation, see Figure 3b). The Motion Cloud object will initialize the specified kernel parameters and run the AR frame generation until the steady state is reached typically using 87 frames for the default parameters. The last three generated frames are stored in the MC object ready for frame-by-frame generation. Callback functions and listeners are used to enable continuous updating of the parameters and, when the GUI is in use, to display the generated stimulus continuously frame by frame. The GUI controls and sliders are all contained within this class. The functionality of the Motion Cloud class is illustrated on the right-hand side of Figure 3.

Two important points about these classes must be noted. First, they are complementary (Dyntex is necessary for Motion Clouds). Second, they cannot simply be called by themselves and expected to generate MCs. Dyntex() will not run as it and must be called within Motion Cloud. Motion Cloud() will initiate a blank Motion Cloud object without generating any frames and set the values in Table 2 to the default ones. The minimal steps needed to generate MC stimuli with these default parameters from the blank object are two internal functions, setParams() to apply the default stimulus parameters from Table 1 and movieDisplay() to initiate the GUI ready to display the motion sequence. This minimal test example is demonstrated in the simpGui script, which is the recommended starting point for new users of the DynTex toolbox. Alternatively, for more control of the initial exploration of the GUI, there is a script called fullGui, which allows more user control of stimulus, technical, and advanced generation parameters from a listed range of defaults. The generation of an object named MC is done within the code so that the object can be saved for offline stimulus generation if required.

**Figure 4.**
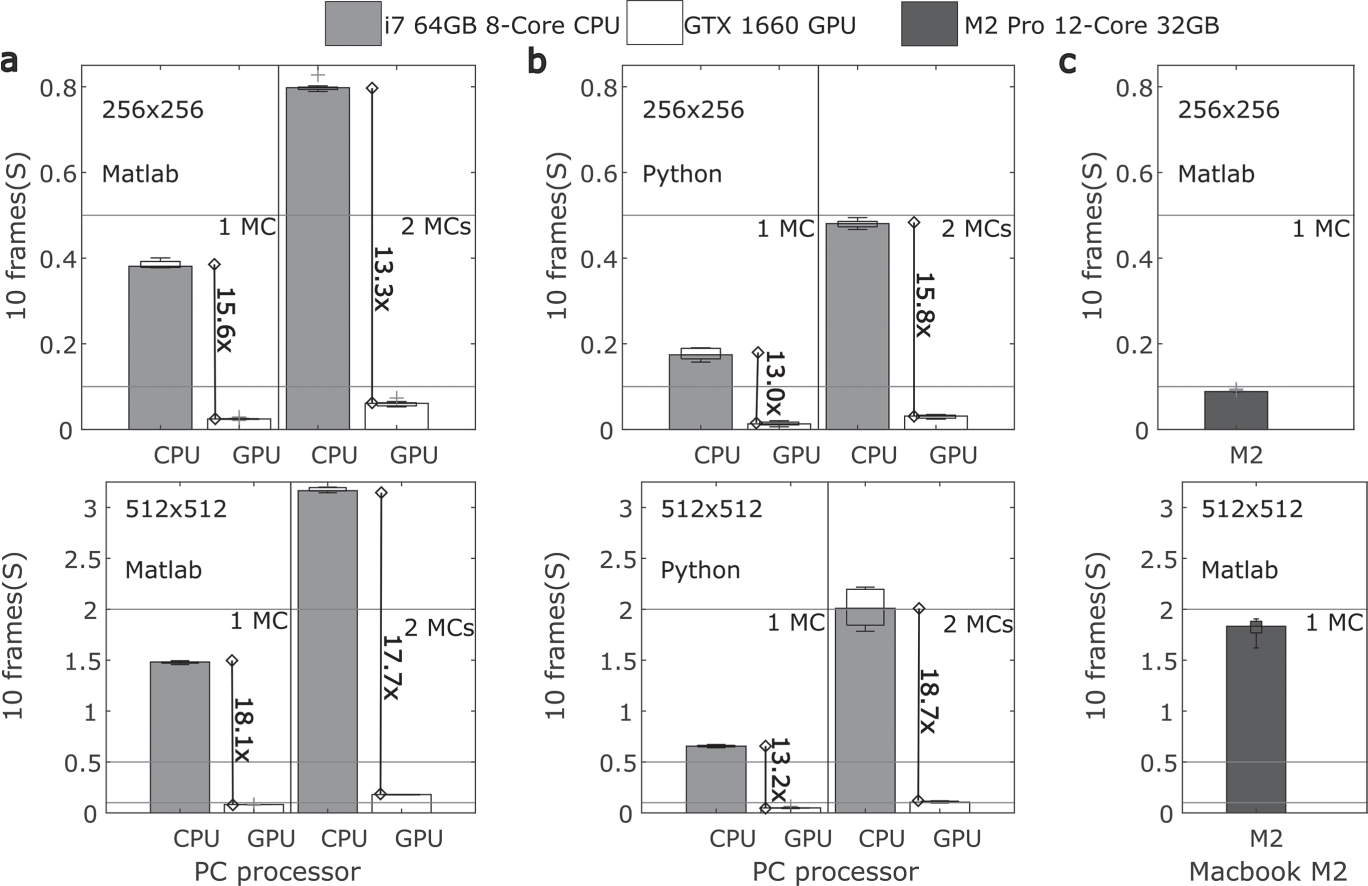
Comparison of the time taken to generate 10 frames of Motion Clouds for a range of processor, image size, and programming language conditions. (a) For MCs generated using MATLAB 2022, total time of generation in seconds is plotted on the ordinate axis for CPU (light gray fill) and GPU (unfilled bar) for a single MC, first two bars, or compounds with two MCs added together in the third and fourth bars along the abscissa. Each bar shows the mean of 10 repetitions, and the overlaid boxplots show the interquartile range. The top row is for a smaller image array of 256 × 256 pixels, while the bottom is a larger array of 512 × 512 pixels. The CPU generation is 13 to 18 times slower than the GPU, shown inset with vertical lines, and reference horizontal lines at 0.1, 0.5, and 2s allow a comparison across all plots. (b) For MCs generated using Python via a Jupyter notebook, the organization of the conditions is identical to that of the first column. Interestingly, generation is faster for both the GPU and CPU. The GPU advantage is still 13 to 19 times faster, with the largest gains for the compound of two MCs. (c) For MCs generated on MATLAB 2022 using the MacBook Pro M2 processor, only the single MC condition (dark gray) is tested for a smaller (top row) and larger (bottom) image sequence. The M2 is about four times faster than the comparable CPU for the smaller image but 40% slower for the larger image. Two computers, a Dell OptiPlex 7090 and a MacBook Pro M2, are used for the generation (see text for details).

As a minimal test of the speed of generation of Motion Clouds, we use a lab computer to measure the duration of computation for (1) a single MC and a composite MC, made up of a pair of stimuli, using (2) both Matlab 2022 and Python as a Jupyter notebook, (3) running on a CPU and GPU and (4) for a smaller 256 × 256 and a larger 512 × 512 image sequence. These manipulations of (1) MC type, (2) programming language, and (3) processor and (4) image size are each repeated 10 times to obtain averages and standard deviations of performance. The manipulation is carried out for each condition by first running the setParams() Motion Cloud function with the default parameters to generate a steady-state set of frames in the object ready to start the synthesis. From this point, 100 frames are generated in a loop using the getFrame() function with an input argument of 0, and the processor clock time for the generation of 10 frames in a fixed position 13 frames from the start is stored. All these manipulations are run on a Dell Optiplex 7090 running Windows 11, which forms part of a laboratory cluster at King’s College London. The PC has an Intel i7-10700 CPU @2.90 GHz, eight cores, and 64 GB RAM. The GPU is an NVIDIA GeForce GTX1660 with 1,408 CUDA cores. A small subset of conditions for the single MC type on MATLAB and for both large and small image sequences is repeated on a Macbook M2 Pro for comparison. The MacBook Pro has an M2 Pro chip with 12 cores (8 performance and 4 efficiency), 32 GB RAM, and no GPU access. The results of these simulations are shown in Figure 4. We find that the CPU computations (in light gray) are consistently 13 to 19 times slower than their GPU counterparts (unfilled bars). GPU computations allow texture synthesis at well under 10 ms per frame (i.e., 1/10 of the duration shown) for both the single MC and the composite pair. This 10-ms threshold, indicated by the lower horizontal line in the figures, would allow for real-time texture synthesis at 100 Hz. For the given computer, Python is also seen to be up to twice as fast as MATLAB for the synthesis, comparing Figures 4a and b. Additional syntheses are generated on the MacBook Pro and compared to the CPU syntheses on the PC (see Figure 4a), with the first gray bars showing that the M2’s performance (Figure 4c) is four times faster for the small images but about 40% slower for the larger images. This suggests that the M2 processors offer efficiency that can be harnessed for smaller matrices, but not for the larger ones.

### MATLAB GUI: The MC visualizer

**Figure 5.**
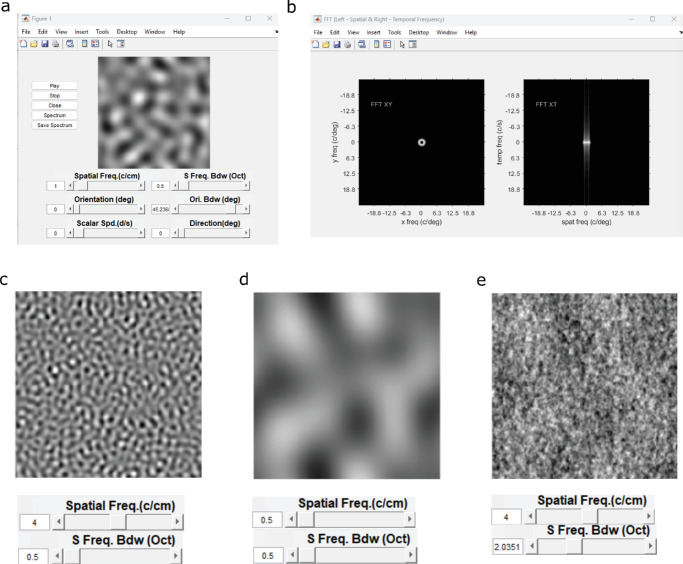
Illustration of the graphic user interface (GUI). (a) An image of the GUI showing the control buttons on the left and parameter sliders below the example of a stimulus frame with a low spatial frequency (1 cycle/cm) and bandwidth (0.5 octaves). (b) The corresponding spatial (left) and spatiotemporal Fourier space profiles of the illustrated stimulus from panel (a). (c) An example stimulus with high spatial frequency (4 cyc/cm) and low bandwidth (0.5 octaves), with the slider corresponding to these parameters. (d) As in panel (c), but with a low spatial frequency (0.5 cyc/cm) and low bandwidth (0.5 octaves). (e) As in panel (d), illustrating a higher bandwidth (2 octaves) with lower spatial frequency (0.5 cyc/cm). Frequencies are approximated for a laptop screen, and generation is done on a MATLAB 2022 system.

A graphic user interface is generated inside the Motion Cloud object by calling the function movieDisplay(). It uses callback functions to interface with the MC object in real time. It therefore acts as a live bridge between the internal object continuously simulating MCs frames on the computer memory and the GUI with a corresponding user visualization of output images and controls of display and parameters. Playing the image sequence on the GUI is controlled by the getFrame() function in a “while” loop before the stop button is pressed. During MC display, spatial frequency, orientation and speed parameters can be adjusted in real time. The GUI is initiated in motion, but using buttons on the left-hand side (see Figure 5a) can be stopped, restarted, or used to view and save the instantaneous Fourier Spectrum of the generated frames, seen in Figure 5b where the low spatial frequency stimulus with isotropic orientation is projected as a doughnut in frequency space. One of the advantages of this GUI is that it can give users a quick intuitive feel for changes in the texture grain as one adjusts mean spatial frequency, for instance, by shifting it from higher to lower values or varying the bandwidth parameters from narrow to broadband. Examples of both changes are illustrated in Figures 5c–e. We recommend that users test the GUI functionality before generating stimuli and running experiments.

### Two visual psychophysics examples

To illustrate the versatility of MC generation and experimental use, the script psychphysDemo presents two examples of demonstration experiments, which showcase some MC properties that have been previously tested. These examples require installing Psychtoolbox with video display libraries.

#### Example 1: A low-level motion task with MCs

The first demo task can be selected by setting DemoRun = 1 at the top of the script. This is a velocity discrimination task in which participants judge the direction of single trials of stimuli in which two factors, speed bandwidths (varying from low to high) and mean speed for two different speeds are manipulated. The parametric manipulations create stimuli in which there are perceptual ambiguities about whether motion is in a rightward or leftward direction. Generation should be carried out on the GPU, and participant keyboard responses and stimulus parameters are stored in the structure S, which is generated by the software and saved on the workspace. This task serves as an illustrative template for the use of MCs in low-level tasks where stimuli are briefly presented for perceptual judgments or ocular tracking responses. For this kind of experiment, two important aspects must be taken into account in the design of the stimuli: (1) which statistical distribution of the reference should be used in a 2 Alternative Forced Choice (2-AFC) comparison when test stimuli are MCs of systematically varied different spatiotemporal mean and bandwidths parameters, and (2) how luminance contrast can be normalized across conditions.

Analogues of classical motion perception tasks such as speed or direction discrimination can be easily implemented and tested using Motion Clouds. For instance, we previously investigated how spatial frequency bandwidth alters speed perception using two different instances of MCs, as illustrated in Figure 2 (Gekas et al., 2017; Simoncini et al., 2012). Motion stimuli were either a single distribution along the speed plane in Fourier space or patterns made by summing three MCs, called components, located at different locations relative to the center of this plane. In both tasks, mean velocity was conserved while spatiotemporal frequency distributions were varied across conditions. We further investigated how the same distributions can affect motion direction discrimination (Pour, Perrinet, Masson, & Montagnini, 2017).

To define the best reference stimulus, in previous research, we tested both a grating (zero frequency variance) or a random dot pattern (broadband variance). We did not observe any significant differences between the two in 2-AFC performance. To control for the stimulus luminance contrast, we normalized MCs using either the Michelson or RMS contrasts across conditions in order to keep motion energy constant (Simoncini et al., 2012). The RMS normalization across different Motion Clouds is done by controlling the pixel value distribution and ensures a match in perceived contrast between Motion Clouds.

Interestingly, the same Motion Cloud stimuli were also used to investigate how human reflexive ocular following responses (Meso et al., 2022; Simoncini et al., 2012) and human voluntary smooth pursuit (Pour et al., 2017) are dependent upon the same MC bandwidth properties. It is therefore possible to compare perception and action using the same naturalistic, well-calibrated stimuli. We found that spatial frequency bandwidth increases resulted in an increase in both initial eye velocity and reliability while they were detrimental for speed perception. The MC statistics can then be used to test a computational model of how motion energy can be integrated or segmented for an optimal estimation of stimulus motion (Gekas et al., 2017; Simoncini et al., 2012)

The earlier studies using motion textures (discussed above) were impeded by the heavy FFT-based computation of images: Movies were always precomputed and loaded into memory in order to randomly interleave their presentation across trials. It was thus impossible to generate new MC instances on each trial. The new AR generation of MC allows to quickly compute complex motion stimuli (see Figure 4). New instances of phase-randomized MCs can then easily be generated on a trial-by-trial basis. Such improvement would allow several experimental advances for low-level tasks such as (1) presenting different instances of the random phase textures, avoiding the repetition of the same random spatial features; (2) using adaptive methods for motion psychophysics in order to reduce the number of trials or design a contingent protocol where a novel stimulus can be defined based on the recent past perceptual (or neuronal) responses; and finally (3) updating and displaying MCs in pseudo-real time over the course of the same trial to probe the effects of a sudden change in, say, orientation bandwidth on either perception or tracking eye movements.

#### Example 2: Investigating perceptual organization with moving textures

Motivated by providing a generic task template for the manipulation of perceptual organization, in our second demo example selected by setting DemoRun = 2 at the top of the script, we implement a perceptual forced-choice task in which participants report whether they perceive one or two directions of motion after each presented trial. In each trial, two MC components with similar spatiotemporal characteristics are superimposed to form a pattern Motion Cloud. Over several trials, the base speed (from slow to fast), spatial frequency (low and high), and motion direction angle are manipulated for the first component. The second component has the same speed and spatial frequency as the first, with the only difference being the angle difference, which goes from 0 (for coherent motion) to 90 degrees. Participants report whether they see one or two motions. This template can be adapted for forced-choice or other tasks involving multiple components. Perceptual reports recorded are stored in the structure S along with all the experimental parameters.

When varying motion energy distributions across different dimensions (i.e., spatial or temporal frequency, orientation, speed or direction) in Motion Clouds, a perceptual transition can occur for very large distribution values: Motion coherence breaks down, and a single MC stimulus can then be perceived as made of multiple components. These perceptual transitions are well known when using classical random dot patterns with different types of additive noise drawn from a distribution of velocity vectors (e.g., Masson, Mestre, & Stone, 1999; Schütz, Braun, Movshon, & Gegenfurtner, 2010), but their impact is often ignored in classical, low-level tasks such as speed or direction discrimination. These effects could be even stronger when adding several Motion Cloud components spanning across the spatiotemporal frequency space (Gekas et al., 2017). MCs therefore offer the strong potential to study this phenomenon of perceptual ambiguity by carrying out controlled parametric manipulations. This would allow researchers to identify the most efficient parameter ranges for driving a given perceptual state (e.g., motion coherence, transparency or other ambiguous states), as well as estimating the transition probabilities between them while still maintaining precise control of statistical distributions along one or several stimulus dimensions. A noteworthy point is that this approach of linking different distribution properties has previously been proposed as a way of understanding optic flow perception, but dimensions other than speed and direction were not easily manipulated for further study (e.g., Andersen, 1989; Andersen & Wuestefeld, 1993; Atchley & Andersen, 1995).

Thanks to the direct control of the energy distributions in naturalistic motion stimuli, it is now possible to probe how spatiotemporal distributions of motion energy influence these perceptual states and their transitions. That is to say, we now have in hand a tool to probe the perceptual organization of moving scenes and therefore interrogate motion segmentation and integration computations more profoundly. Previously, these questions were addressed indirectly through changes in speed or direction discrimination and measured changes in sensitivity ranges of different secondary perceptual judgments (see Curran & Braddick, 2000; Mestre & Masson, 1997). These perceptual states were also previously studied with subjective methods (e.g., Andersen & Wuestefeld, 1993), but mostly if not solely at the level of velocity flow fields (see Nishida et al., 2018). By manipulating the statistical properties across these multiple dimensions precisely, we can generate a set of stimuli that can be linearly spaced along one or several physical dimensions of the images/textures. By doing so, we can probe how they are perceived using forced-choice procedures such as maximum likelihood difference scaling (MLDS) and maximum likelihood conjoint measurement (MLCM) (Maloney & Knoblauch, 2020).

#### The future: From trial-based to continuous psychophysics

It has recently been proposed that when dynamic and adaptive adjustment of stimulus parameters are possible, experimenters can measure sensitivity in naturalistic settings with a very efficient method called continuous psychophysics (Bonnen, Burge, Yates, Pillow, & Cormack, 2015; Falconbridge, Stamps, Edwards, & Badcock, 2023). This interesting new approach requires stimuli to be generated fast and adjusted within tens of milliseconds while responses are being obtained, in contrast to the more classical trial-by-trial paradigms. The continuous measurements can then be analyzed to separate sensory sensitivity from other factors that affect tracking performance (Straub & Rothkopf, 2022). Both task examples shown above follow the classic psychophysics paradigm with stimulus conditions preset and presented over the course of several randomized trial presentations. As stimuli are generated before each trial, any parameters specified by the variable “kernel params” structure can be updated at the start of each trial to adapt to previous responses, for example, when using staircase procedures. The coding of the GUI also provides a template for presentation in which the response of a single trial can be changed over time. The movieDisplay function does a real-time update of the current Motion Cloud on the figure object, and this could be done on a display screen while using callbacks to update parameter values. Therefore, the present toolbox offers the opportunity to use the continuous psychophysics method with Motion Clouds.

## Discussion

The legacy of visual neurosciences and visual perception studies has been the bouncing back-and-forth between using artificial, low-dimension stimuli such as gratings or random dots (Lorenceau, 1996; Lorenceau & Gorea, 1984) or high-dimension inputs, such as natural images (Carandini et al., 2005). In their advocacy for artificial, well-controlled stimuli, Rust and Movshon (2005) called for new classes of synthetic stimuli that are both entirely parametrized and can mimic the statistics of natural images. Motion Clouds are one instance of such a new, naturalistic stimulus (Leon et al., 2012). They fulfill these two criteria. Built from a family of Gabor-like patterns whose properties can be fully parametrized (i.e., size, location, and spatiotemporal frequency content), MCs can be generated by controlling the statistical properties (i.e., mean and variance) of motion energy distributions along several dimensions: spatial and temporal frequency, orientation/direction and sparseness. Local geometry (i.e., the phase of each Gabor instance) is randomized so spatial patterns generated and set into motion produce unique flow fields for each instance. Lastly, several MCs can be weighted according to their contrast and added to generate complex MC patterns from individual MC components.

The proper usage of any new class of visual stimuli relies on the existence of documented and freely distributed toolboxes that can be easily integrated into the major experimental packages. DynTex easily fits into the two major packages used in visual neurosciences: Psychtoolbox and PsychoPy. By allowing offline stimulus generation and storage, it is also possible to use any experimental package that can upload image frames. DynTex is documented in this article so that users can easily generate new stimuli but also understand the exact properties of outputs. Gratings and grating-based patterns (plaids, multiple Gabor micropatterns, and others) are popular because they easily meet these two criteria of being user-friendly and tractable. In this work, we sought to present a toolbox that did the same for our naturalistic stimuli, Motion Clouds.

We believe that this documented toolbox, in the hands of our colleagues, has the potential to facilitate empirical and theoretical work that could have far-reaching consequences within and beyond the visual psychophysics community. Indeed, Motion Clouds can be used in many different fields that traditionally rely on tasks using controlled visual stimulation. For example, within the field of cross-modal and multisensory perception, Rezk et al. (2020) compared representations of auditory and visual motion direction in human middle temporal cortex. In such findings, the use of MCs, and their equivalent in the auditory domain, could support further systematic manipulation of speed, direction, and coherence, potentially leading to a more fine-grained understanding of how different motion parameters are integrated across senses. In the field of developmental psychology, MCs could serve as a valuable tool for investigating motion integration and segmentation at different stages of development, as well as the specific aspects of motion perception affected in dyslexia, such as the potential common underlying mechanisms linking motion-processing deficits and dyslexia (Joo, Donnelly, & Yeatman, 2017). In the field of cognitive and perceptual aging, MCs could be utilized to explore age-related changes in motion perception and serve as a conceptual framework for perceptual aging, as highlighted by Billino and Pilz (2019). Specifically, our extensive work with MCs and ocular following responses could be a starting point for investigating age-related differences in performance. This would complement findings on smooth pursuit eye movements and the observed decline in basic motor parameters, in contrast to the stability found in prediction and anticipation of target motion (Sprenger et al., 2011).

In this article, we have have set up a unified resource for colleagues who might consider using Motion Clouds in their research. Key research findings from the fields of visual perception, eye movements, neurophysiology, computational neuroscience, and applied mathematics that were based on Motion Clouds were discussed. These advances were typically possible because of spatiotemporal frequency properties, which could be precisely manipulated in the stimuli. By discussing the two different algorithmic approaches for generating Motion Clouds, the FFT (Leon et al., 2012) and AR2 (Vacher et al., 2018) methods, the speed advantages of the latter, particularly when using GPUs, were put into context. The DynTex toolbox is available online under the links provided in MATLAB and Python. The authors of this article will continue to use and develop DynTex, and we hope to build a wider community that will contribute to future development.

## Figures and Tables

**Figure A1.**
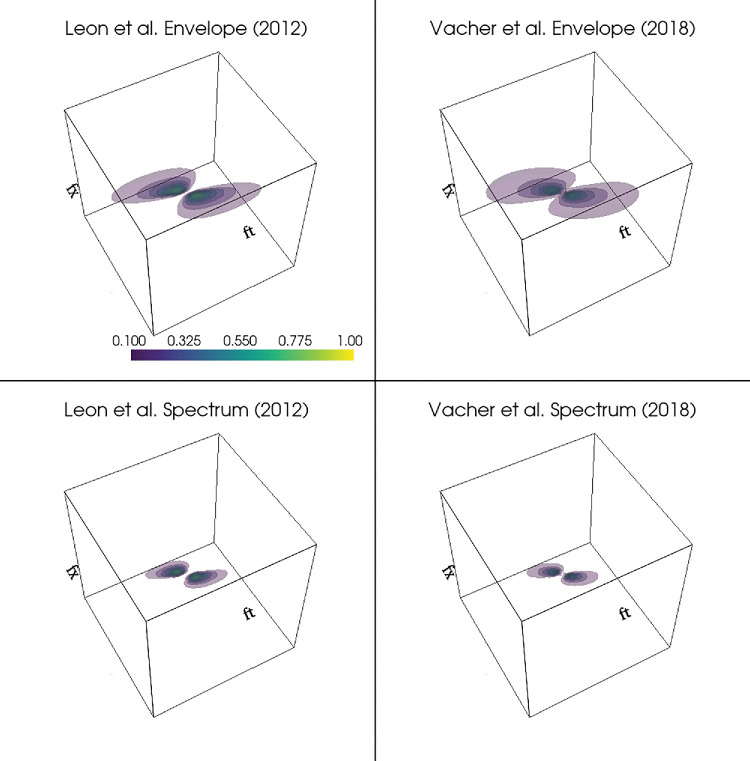
Examples of the envelope (top row) and power spectrum (bottom row) obtained with Leon’s FFT (left column) and Vacher’s AR2 (right) generation. Parameters have been manually adjusted so that both methods lead to comporable envelope and power spectra.

## Appendix: Comparing FFT (2012) to AR2 (2018) MC generation

In both versions, MCs are defined by their power spectrum $\hat{\gamma }$ or its corresponding envelope E (which is the square root of the power spectrum). In Leon et al. (2012), which involves the fast Fourier transform (FFT) of the full stimulus spatiotemporal cube, the envelope is defined as a product of densities over radial frequencies, their orientation, an assumed speed density, and a spectral color scaling factor. In contrast, in Vacher et al. (2018), which is generated using an autoregressive process (AR2), the power spectrum is derived theoretically from the definition of MCs as the random aggregation of warped patterns. The resulting power spectrum is the product of the densities over the radial frequencies, their orientation, and a transformation of the speed density. The major difference concerns the speed density; for the FFT generation of Leon et al. (2012), the choice was made following empirical observations (Dong & Atick, 1995) of the power spectrum of natural movies, while in the AR2 generation by Vacher et al. (2018), it is the result of a theoretical derivation. The two sections below summarize the key equations that define the envelope and the power spectrum. Table A1 summarizes the different notations that were used, and Figure A1 displays the isosurfaces of both the power spectrum and envelope obtained with both definitions showing that they lead to similar results.

**Table A1.** Comparison of parameterization between Leon’s FFT and Vacher’s AR2 models with location and scale parameter types. Note that the parameter sf_bdw controls the bandwidth in both linear and log scale depending on the value of octa (0/1) within the code.

		Scale	
Parameter	Location	Linear	Log
Speed			
Leon et al. (2012)	*V*_*x*_, *V*_*y*_	–	*B*_*V*_
Vacher et al. (2018)	$v_{0}\in R^{2}$	σ_*V*_	–
DynTex	speed	speed_sig	–
Orientation			
Leon et al. (2012)	θ	*B*_θ_	–
Vacher et al. (2018)	θ_0_ (mean)	σ_θ_	–
DynTex	th	th_sig	
Spatial Frequency			
Leon et al. (2012)	*f*_0_	–	*B*_*f*_
Vacher et al. (2018)	*z*_0_	σ_*Z*_	*B*_*Z*_
DynTex	sf	sf_bdw	sf_bdw

### FFT generation of Leon and colleagues

The equations presented here are updated compared to the original publication so that they correspond to the legacy implementation available on GitHub.^1^ The envelope with velocity, spatial frequency, orientation, and spectral color terms, E, is defined as follows:

(8) $$E=V_{\left(V_{x},V_{y},B_{V}\right)}G_{\left(f_{0},B_{f}\right)}O_{\left(\theta ,B_{\theta }\right)}C_{\left(\alpha \right)}.$$

These last three factors of the envelope expanded in full are

(9) $$\begin{array}{ccc} & & \;G_{\left(f_{0},B_{f}\right)}\left(f_{x},f_{y},f_{t}\right)=\frac{1}{f_{r}}\;\exp\left(-\frac{1}{2}{\left(\frac{\ln\left(\frac{f_{r}}{f_{0}}\right)}{\ln\left(\frac{f_{0}+B_{f}}{f_{0}}\right)}\right)}^{2}\right),\\ & & \;O\left(f_{x},f_{y}\right)=\exp\left(\frac{\cos(\theta_{f}-\theta )}{B_{\theta }}\right),\;C_{\alpha }\left(f_{x},f_{y},f_{t}\right)=\frac{1}{f_{r}^{\alpha }}\; \end{array}$$

where $f_{r}=\sqrt{f_{x}^{2}+f_{y}^{2}}$ is the radial frequency, θf= atan 2(fx,fy) is the angle in the Fourier domain, and α ∈ [0, 2]. Moreover, the speed envelope is

(10) $$\begin{array}{ccc} & & \;V_{\left(V_{x},V_{y},B_{V}\right)}\left(f_{x},f_{y},f_{t}\right)\\ & & \;\;=\exp\left(-\frac{1}{2}{\left(\frac{f_{x}\cdot V_{x}+f_{y}\cdot V_{y}+f_{t}}{B_{V}\cdot f_{r}}\right)}^{2}\right).\; \end{array}$$

As a consequence, changing the speed of a texture results in warping the envelope in frequency space along the temporal frequency axis, while the projection of the envelope on the two spatial frequency dimensions remains unchanged.

### AR2 generation of Vacher and colleagues

The equations below are the ones presented in the original work, and they correspond to those used in the proposed DynTex toolbox. The power spectrum $\hat{\gamma }$, with parameterized distributions of spatial frequency, orientation, and speeds, is defined as follows:

(11) $$\begin{array}{ccc} & & \;\forall \;\left(\xi ,\tau \right)\in R^{2}\times R,\;\hat{\gamma }\left(\xi ,\tau \right)\\ & & \;=\frac{P_{Z}\left(|\;|\xi |\;|\right)}{{\left|\;|\xi |\;\right|}^{2}}P_{\Theta }\left(∠\xi \right)L\left(P_{|\;|V-v_{0}\left|\;\right|}\right)\left(-\frac{\tau +\langle v_{0},\;\xi \rangle }{\left|\;|\xi |\;\right|}\right),\; \end{array}$$

where $\left|\;|\xi |\;\right|=\sqrt{\xi_{1}^{2}+\xi_{2}^{2}}$, ∠ξ= atan 2ξ1,ξ2, $v_{0}\in R^{2}$ and with

(12) $$\forall \;u\in R,\;L\left(f\right)\left(u\right)\overset{\mathrm{def}.}{=}\int_{-\pi }^{\pi }f\left(-u/\cos\left(\varphi \right)\right)d\varphi ,$$

and with the following densities and speed transformation (enforced by the autoregressive AR2 implementation)

(13) $$\begin{array}{ccc} & & P_{Z}\left(z\right)\propto \frac{1}{z}\exp\left(-\frac{\ln{\left(\frac{z}{{\tilde{z}}_{0}}\right)}^{2}}{2\ln\left(1+{\tilde{\sigma }}_{Z}^{2}\right)}\right),\\ & & P_{\Theta }\left(\theta \right)\propto \exp\left(\frac{\cos(2\left(\theta -\theta_{0}\right))}{4\sigma_{\Theta }^{2}}\right),\\ & & L\left(P_{|\;|V-v_{0}\left|\;\right|}\right)\left(u\right)\propto \frac{1}{{\left(1+\frac{u^{2}}{\sigma_{V}^{2}}\right)}^{2}}. \end{array}$$
